# O23 Pulmonary emboli in a teenager with GPA: management dilemmas

**DOI:** 10.1093/rap/rkaa054.011

**Published:** 2020-11-03

**Authors:** Sam Deepak, Satyapal Rangaraj, Kishore Warrier

**Affiliations:** Nottingham Children’s Hospital, Queens Medical centre, Nottingham, United Kingdom

## Abstract

**Case report - Introduction:**

15 year old girl with the diagnosis of granulomatosis polyangiitis (GPA) managed with induction regimen of intravenous cyclophosphamide and whilst on maintenance mycophenolate mofetil (MMF) developed multiple cavitating lung lesions with the large cavity abutting pulmonary vein and bilateral segmental pulmonary embolism (PE) posing complex management dilemmas.

**Case report - Case description:**

15-year-old girl presented with being unwell for 3 months with malaise, lethargy, joint pains, significant weight loss (10 kg), mouth ulcer and significant hearing loss. Investigations showed anaemia, raised inflammatory markers, and impaired kidney function (estimated glomerular filtration rate eGFR 40). Her ANCA was positive, hearing test showed significant mixed hearing loss and CXR was normal. The renal biopsy confirmed pauci-immune ANCA associated glomerulonephritis with 70% crescents. She was initially managed with intravenous pulse of steroids followed by oral weaning regime, double filtration plasmapheresis and commenced on induction regimen of intravenous cyclophosphamide. She received 6 doses of cyclophosphamide 500 mg/m2 and following good recovery with normalising kidney function; was commenced on maintenance MMF.

At this point she developed new onset earache, sore throat, and hoarseness of voice with raised inflammatory markers and worsening symptoms despite antibiotics. This was presumed to a flare of vasculitis and hence was given further pulse of steroids and increased the dose of MMF. The ENT assessment did not reveal any subglottic stenosis. After few weeks, symptoms recurred with cough/hoarseness of voice and associated tiredness. Bloods showed raised inflammatory markers; CXR revealed cavitating lung lesions and a CT chest was arranged.

CT chest showed apical sub pleural lung nodule and a large thick-walled cavity measuring 6.6x 4.4 cm abutting the pulmonary vein on the right side and bilateral segmental pulmonary emboli. The child was systemically stable with no respiratory distress and oxygen saturations were 100% in air.

**Case report - Discussion:**

The management of GPA was further complicated by the pulmonary embolism and cavitating lung lesions abutting pulmonary vein.

The management included escalation of immunosuppression with pulse of steroids, further dose of cyclophosphamide and commence Rituximab .The key challenges with the immediate management were risk of bleeding associated with the anticoagulation, treating the pulmonary embolism, risk of diffuse alveolar haemorrhage and managing the patient in a safe setting equipped with all the expertise required.

The child was screened for cardiolipin antibodies on multiple occasions and these were negative. An ECHO was done to look for evidence of clot at the end of central line tip, but this was normal. Deep venous thrombosis of legs was ruled out by Doppler scanning. There was no clear source of emboli identified.

Although there is emerging evidence for increased incidence of vascular events in GPA adult patients, the data on vascular events in children with GPA is scarce. Merkel and co-workers reported a high occurrence of pulmonary embolism (PE) and deep venous thrombosis (DVT) among GPA patients included in a randomized therapeutic trial (WeCLOT study) ^1^ .FAURSCHOU et al. reported that GPA was associated with a much lower relative risk of stroke than of pulmonary embolism and deep venous thrombosis; the risk of venous thromboembolic events among GPA patients was increased during early as well as late follow up periods. Currently there are no significant data on the use of antiplatelet and/or anticoagulant therapy in AAV. Following extensive multidisciplinary discussion with respiratory, haematology, cardiology, cardiothoracic surgical and paediatric intensive care teams, and the child was anticoagulated with close monitoring in paediatric high dependency unit and immunosuppression escalated alongside.

**Case report - Key learning points**
 

1. This case highlights the risk of thromboembolic events in children with GPA
2. Proposed mechanisms in the literature for thrombosis in vasculitis at molecular level would probably explain the episode in the absence of source identified
3. Multidisciplinary team approach is crucial for management of complex patients
4. There were few challenges due to geographical location of the patient and the regional variation of subspecialty cover provided for their local District General Hospital
5. For discussion- Role of Rituximab early in GPA?

